# Safety and metabolic effects of tesamorelin, a growth hormone-releasing factor analogue, in patients with type 2 diabetes: A randomized, placebo-controlled trial

**DOI:** 10.1371/journal.pone.0179538

**Published:** 2017-06-15

**Authors:** David R. Clemmons, Sam Miller, Jean-Claude Mamputu

**Affiliations:** 1 Division of Endocrinology, Department of Medicine, University of North Carolina at Chapel Hill, Chapel Hill, North Carolina, United States of America; 2 SAM Clinical Research Center, San Antonio, Texas, United States of America; 3 Theratechnologies Inc., Montreal, Quebec, Canada; Florida International University Herbert Wertheim College of Medicine, UNITED STATES

## Abstract

**Objective:**

Use of growth hormone is associated with side effects, including insulin resistance. The objective of this study was to determine whether tesamorelin, a stabilized growth hormone-releasing hormone analogue, would alter insulin sensitivity or control of diabetes.

**Design:**

A 12-week randomized, placebo-controlled study of 53 patients with type 2 diabetes. Three treatment groups: placebo, 1 and 2 mg tesamorelin.

**Measurements:**

Fasting glucose, glucose and insulin from oral glucose tolerance test, glycosylated hemoglobin (HbA_1c_), home blood glucose, insulin-like growth factor-1, and lipids.

**Main outcome measure:**

Relative insulin response following oral ingestion of glucose.

**Results:**

No significant differences were observed between groups in relative insulin response over the 12-week treatment period. At Week 12, fasting glucose, HbA_1c_ and overall diabetes control were not significantly different between groups. In addition, relevant modifications in diabetes medications were similar between groups. Total cholesterol (-0.3±0.6 mmol/L) and non-HDL cholesterol (-0.3±0.5 mmol/L) significantly decreased from baseline to Week 12 in the tesamorelin 2 mg group (p<0.05 vs. placebo). No patient discontinued the study due to loss of diabetes control.

**Conclusions:**

Treatment of type 2 diabetic patients with tesamorelin for 12 weeks did not alter insulin response or glycemic control.

**Trial registration:**

ClinicalTrials.gov NCT01264497.

## Introduction

Recombinant human growth hormone (rhGH) replacement therapy has been shown to reverse several metabolic alterations associated with low serum GH level or GH deficiency, including increased visceral adipose tissue (VAT), altered lipid profile, and impaired physical performance [1–3]. However, administration of pharmacological doses of rhGH is associated with a variety of adverse effects, including hyperglycemia, insulin resistance, fluid retention, and carpal tunnel syndrome [3–4]. In contrast, strategies using growth hormone-releasing factor (GRF)/growth hormone-releasing hormone (GHRH) analogues to induce physiological increases of GH and preserve the insulin-like growth factor-1 (IGF-1) negative feedback have been shown to correct metabolic abnormalities and body composition changes associated with low GH levels with fewer side effects, particularly with regard to hyperglycemia [5–8].

Tesamorelin (Theratechnologies, Inc., Montreal, Quebec, Canada) is a synthetic analogue of human GHRH that increases basal and pulsatile secretion of GH [9–10]. A hexenoyl moiety has been anchored at the N-terminus of the 44 amino acid sequence of human GHRH, resulting in enhanced stability in serum as compared to natural human GHRH. Because GH has been shown to worsen glucose tolerance in some subjects, including those receiving highly active antiretroviral therapy (HAART) [11], a randomized, parallel, placebo-controlled, multicenter study was carried out to assess the safety of tesamorelin in only subjects with type 2 diabetes mellitus.

## Methods

The protocol for this study and supporting CONSORT checklist are available as supporting information: see S1 CONSORT checklist and S1 Protocol.

### Study design

**Fig 1:**
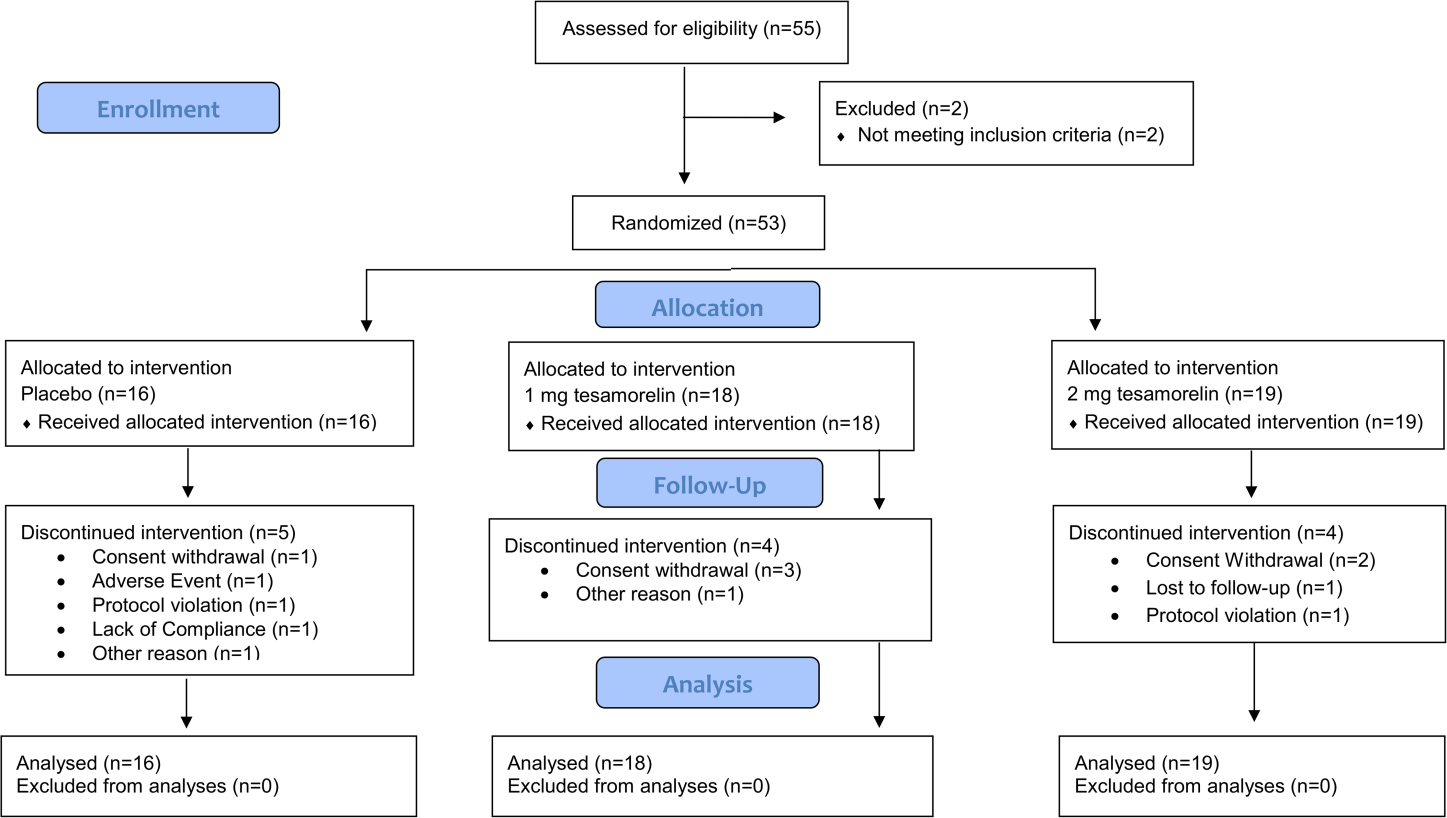
CONSORT flow diagram of the clinical study. Patient enrollment and outcomes.

Patients with stable type 2 diabetes were recruited at 5 sites in the US between February and November 2002. After screening and a 14-day lead-in period to establish and characterize daily dietary patterns and blood measurements, patients diabetes were randomly assigned to receive either placebo, tesamorelin 1 mg, or tesamorelin 2 mg, administered by subcutaneous injection for 12 weeks (Fig 1). The study included a 4-week follow-up period to monitor for adverse events. Randomization was stratified on the basis on insulin use. To maintain balance of treatment assignments among insulin use groups (insulin use, no insulin use), eligible patients were randomly allocated in balanced blocks (1:1:1 ratio) to one of the 3 treatment groups according to computer generated randomization codes, which were prepared by a statistician who was not otherwise involved in the study. Patients and investigators were unaware of assignments to study groups. Tesamorelin and matching placebo were provided as lyophilized powder for reconstitution in sterile water. Inclusion criteria included: male or postmenopausal or surgically sterilized female subjects, ≥50 years of age; diagnosis of type 2 diabetes for ≥3 months before screening; on stable diabetes treatment regimens (oral hypoglycemics with or without insulin) for ≥2 months before screening; screening and pre-randomization HbA_1c_ <10% (baseline range: 5.3–9.5%) and body mass index (BMI) between 25 and 38 kg/m^2^. Exclusion criteria included: positive mammography (if female) or prostate-specific antigen (PSA) or prostate examination for cancer (if male); use of oral or parenteral glucocorticoids in the 30 days before screening; use of any experimental or marketed GH, GH secretagogue, IGF-1, or insulin-like growth factor binding protein-3 (IGFBP-3) in the previous 6 months; history of or presence of active concomitant conditions or diseases; documented hypopituitarism, history of pituitary tumor/surgery, head irradiation, or severe head trauma; and current or history of cancer.

### Metabolic measurements

Fasting blood samples were drawn at baseline and at Weeks 1, 4, 8, and 12 of treatment for measurement of fasting glucose and IGF-1 levels. Samples were drawn at baseline and at Weeks 8 and 12 for determination of triglyceride, total cholesterol, low-density lipoprotein (LDL) cholesterol, and high-density lipoprotein (HDL) cholesterol levels. Fasting blood samples were drawn at baseline and at Weeks 4, 8, and 12 for determination of glycosylated hemoglobin (HbA_1c_) level. An Oral Glucose Tolerance Test (OGTT) using 75 grams of glucose was performed at baseline and after 1, 4, 8, and 12 weeks of treatment. The subjects had fasted overnight prior to the OGTT. Paired insulin and glucose measurements were performed at 0 minute (before ingestion of 75 grams of glucose) and every 30 minutes during the OGTT. Patients were instructed to withhold insulin and sulfonylurea hypoglycemic medications on the days in which OGTT was performed. Insulin sensitizers were not withheld. Home blood glucose was obtained four times per day (before breakfast, lunch, and dinner, and at bedtime) using a standard blood glucose meter. The glucose values were downloaded into a computerized recall system. The measurements were used to calculate mean daily blood glucose values. In addition, the number of dose adjustments per week for insulin and/or oral hypoglycemic agents, as well as control of diabetes, was recorded.

### Laboratory methods

All metabolic parameters were measured centrally at Esoterix Inc. (Calabasas Hill, CA). Fasting glucose and insulin, HbA_1c_, total cholesterol, HDL cholesterol, LDL cholesterol, and triglycerides were determined using standard laboratory assays. Plasma IGF-1 was measured using radioimmunoassay (RIA) kits, with a lower limit of detection of 10 ng/mL.

### Body composition measurements

Anthropometric measurements were determined by standard techniques. Body weight (BW) was measured to the nearest 0.1 kg and height (H) to the nearest millimeter. Body mass index (BMI) was calculated as BW (kg) divided by H (m^2^). Waist circumference was measured in the standing position midway between the lower rib margin and the iliac crest using a tape measure. Body fat was estimated from measurements with Lange skin-fold calipers at biceps, triceps, subscapular, and iliac crest sites, as previously described [12].

### Compliance

Subjects recorded their compliance with the treatment regimen in diaries. Compliance was checked by counting the number of empty vials returned at each visit.

### Statistical analyses

The primary endpoint of the study was the relative insulin response over the 12-week treatment period. Relative insulin response was defined as the ratio of the incremental response in plasma insulin to that of plasma glucose during the first 30 minutes of the OGTT, divided by basal insulin, i.e., [delta Insulin (30 min- 0 min)/delta glucose (30 min- 0 min)]/basal insulin, as previously described [13].

Secondary endpoints included changes from baseline to Week 12 in HbA_1c_, changes in mean daily blood glucose concentrations compared to values from the 14-day lead in period, as well as changes in the number of dose adjustments per week for insulin and/or oral hypoglycemic agents, and the number of patients with a change in the control of diabetes.

Sample size was planned to assess a 15% change in the relative insulin response in each of the treatment groups and a constant standard deviation of 0.20, based on data from a previous study involving diabetic subjects [14]. Statistical analyses were performed by Charles River Laboratories Clinical Services (Wilmington, NC, USA) using SAS statistical software (SAS Institute Inc., Cary, NC). Additional parametric statistical analyses were performed by Excelsus Statistics Inc. (Montreal, Quebec, Canada). Baseline characteristics were compared using Fisher’s Exact test for categorical variables and linear mixed effect models for continuous variables. Changes from baseline in relative insulin response and HbA_1c_ were compared among the treatment groups using a repeated-measures analysis of variance (ANOVA) model with treatment, insulin use, and study center as between-subject main effects and time as within-subject main effect. The within-subject random errors associated with repeated-measures across time were modeled using the covariance structure that minimizes the Aikaki’s criterion (AICC). The same approach was used for anthropometric and metabolic parameters. Glucose and insulin levels from the OGTT (change from pre-ingestion) were analyzed using a repeated-measures analysis of covariance (ANCOVA) model with pre-ingestion at baseline (Week 0) as the covariate, with treatment, insulin use, study center, Week (Weeks 1, 4, 8, 12) and Time Point (30, 60, 90, 120, 150 and 180 minutes) as factors and subject as random effect. Main daily home blood glucose change from baseline was analyzed using a repeated measures ANOVA model with treatment, insulin use, study center, Week (1 to 14) and time of the day (Breakfast, Lunch, Dinner and Bedtime) as factors and subject as random effect. A simple within-subject correlation was chosen for those analyses because of the complexity of the two-level time variable (Weeks and Time points/Time of the day). The graphical analysis of the residuals indicated that this model was appropriate. Unless otherwise specified, all statistical tests were two-sided at a 0.05 significance level without corrections for multiple outcomes or multiple comparisons. The primary treatment comparison was the comparison of the two doses (1 mg and 2 mg tesamorelin). The safety population, which was defined as all subjects who were randomized and received at least one dose of study medication, was the primary population for analysis. As the primary objective of the study was to assess the safety of tesamorelin, no method was performed to impute missing data in the primary analysis of the primary endpoint.

### Ethics statement

The protocol of this study was approved by the institutional review board (IRB) used by the participating sites. Three sites used the Western Institutional Review Board [WIRB] (original approval date: January 16, 2002) and two sites used local IRBs: the MRI Institutional Review Board at Washington Hospital Center (original approval date: January 17, 2002) and the Institutional Review Board for Health Science Research at the University of Virginia (original approval date: May 22, 2002). All patients provided institutionally approved, written informed consent. The study was registered at clinicaltrials.gov (NCT01264497). There was a delay in study registration due to administrative oversight. The authors confirm that all ongoing and related trials for this drug/intervention are registered.

## Results

### Subject baseline characteristics and disposition

Of fifty five (55) patients assessed for eligibility, 53 received at least one dose of study treatment and were included in the analyses (16 patients randomized to placebo, 18 to tesamorelin 1 mg, and 19 to tesamorelin 2 mg). The proportion of patients completing the study was not statistically significantly different among the placebo (69%), tesamorelin 1 mg (78%), and tesamorelin 2 mg (79%) groups. No patients withdrew from the study due to loss of diabetes control. Patient enrollment and outcomes are shown in Fig 1.

As shown in Table 1, there were no significant differences among treatment groups in demographics or baseline characteristics, except for a statistically significant higher mean fasting insulin level in the tesamorelin 1 mg group. Most patients (74%) did not use insulin, while 26% were insulin users.

**Table 1:** Demographics and baseline characteristics of the patients.

	Placebo (N = 16)	Tesamorelin 1mg (N = 18)	Tesamorelin 2mg (N = 19)	*P* value
Age (years)	63.1±7.0	59.5±7.9	60.3±6.7	0.40
Gender (n, %)				
Male	12 (75)	9 (50)	14 (74)	0.22
Female	4 (25)	9 (50)	5 (26)	
Race (n, %)				
Caucasian	12 (75)	7 (39)	10 (53)	0.25
Hispanic/Latino	3 (19)	7 (39)	6 (32)	
Asian/Pacific Islander	0	3 (17)	3 (16)	
Other	1 (6)	1 (6)	0	
Weight (kg)	91±15	91±16	95±18	0.64
BMI (kg/m^2^)	30.3±4.2	32.2±4.2	32.1±4.2	0.32
Fasting glucose (mmol/L)	6.9±2.5	8.7±2.4	7.7±1.4	0.09
Fasting insulin (pmol/L)	86±41	168±12	100±68	0.02
Relative Insulin Response (L/pmol)	0.26±0.27	0.15±0.27	0.07±0.45	0.34
HbA_1c_ (%)	6.8±0.9	7.4±0.9	6.9±0.7	0.16
Insulin status (n, %)				
Users	4 (25)	5 (28)	5 (26)	>0.99
Non-users	12 (75)	13 (72)	14 (74)	

Abbreviations: BMI, body mass index. Data are presented as mean ± SD unless otherwise specified.

### Relative insulin response

Mean relative insulin response following oral ingestion of glucose increased slightly from baseline in all groups at all time points over the 12-week treatment period. There were no significant differences between groups in treatment or time effect (Table 2). At Week 12, changes from baseline in mean relative insulin response were 0.02±0.08, 0.05±0.37, and 0.13±0.56 L/pmol for the placebo, tesamorelin 1 mg, and tesamorelin 2 mg groups, respectively. The relative insulin response for most patients, however, remained below mean values reported for normoglycemic individuals (1.85 ±1.18 L/mmol) (data not shown).

**Table 2:** Changes from baseline in relative insulin response (L/pmol) over the 12-week treatment period.

	Placebo (N = 16)	Tesamorelin 1mg (N = 18)	Tesamorelin 2mg (N = 19)
**Baseline**	0.26 (0.27)	0.15 (0.27)	0.07 (0.45)
**Week 1**			
Change from baseline	0.06 (0.34)	0.00 (0.28)	0.16 (0.58)
**Week 4**			
Change from baseline	0.03 (0.26)	0.08 (0.21)	0.15 (0.45)
**Week 8**			
Change from baseline	0.10 (0.33)	0.12 (0.17)^b^	0.11 (0.48)
**Week 12**			
Change from baseline	0.02 (0.08)	0.05 (0.37)	0.13 (0.56)
P-value Treatment^a^	0.71		
P-value Week	0.80		

Data are reported as mean (SD)

^a^P-values from a repeated mesures ANOVA on relative insulin response with main effects for treatment, insulin use (Yes/No), week and center. Within-patient covariance was modeled using the Unstructured covariance structure.

### Glucose levels during the OGTT

Glucose levels during the OGTT were not significantly different between the treatment groups over the 12-week treatment period. As shown in Table 3, changes in glucose levels at 30 and 120 minutes post-glucose load during the OGTT were similar between the 3 groups at Weeks 0 (baseline), 1, 4, 8, and 12. At Week 12, the post-glucose load values at 120 minutes were 7.8±2.9, 6.7±3.2, and 8.7±3.6 mmol/L for the placebo, tesamorelin 1 mg, and tesamorelin 2 mg, respectively. As expected with the OGTT, glucose levels increased in all the treatment groups 30 and 60 minutes following ingestion of the 75-g glucose load.

**Table 3:** Glucose levels (mmol/L) pre-ingestion (0 minute) and changes from pre-glucose ingestion values at 30 and 120 minutes during the OGTT.

	Placebo (N = 18)	Tesamorelin 1mg (N = 19)	Tesamorelin 2mg (N = 16)
**Week 0 (Baseline)**			
Pre-glucose ingestion	6.9 (2.4)	8.4 (2.2)	7.6 (1.4)
Change at 30 minutes	5.6 (2.1)	4.9 (2.0)	5.1 (2.7)
Change at 120 minutes	7.4 (2.3)	7.2 (2.6)	8.9 (1.9)
**Week 1**			
Pre-glucose ingestion	7.0 (1.6)	9.0 (2.4)	8.0 (2.0)
Change at 30 minutes	5.2 (1.9)	5.2 (2.3)	5.5 (2.1)
Change at 120 minutes	6.8 (2.5)	6.8 (2.5)	8.5 (2.2)
**Week 4**			
Pre-glucose ingestion	6.6 (1.7)	9.1 (2.5)	8.5 (1.7)
Change at 30 minutes	5.9 (2.8)	4.7 (2.3)	5.6 (1.0)
Change at 120 minutes	6.5 (2.2)	7.5 (3.8)	8.6 (2.3)
**Week 8**			
Pre-glucose ingestion	7.2 (1.9)	7.8 (1.9)	8.2 (1.5)
Change at 30 minutes	5.9 (2.9)	5.0 (1.7)	5.5 (2.3)
Change at 120 minutes	8.1 (3.4)	6.3 (3.4)	8.2 (2.5)
**Week 12**			
Pre-glucose ingestion	6.9 (1.7)	8.0 (2.2)	7.7 (2.4)
Change at 30 minutes	5.4 (2.7)	5.0 (1.6)	5.6 (1.9)
Change at 120 minutes	7.8 (2.9)	6.7 (3.2)	8.7 (3.6)
P-value Treatment^a^	0.28		
P-value Week^a^	0.28		
P-value Time Point^a^	<0.001		

Data are reported as mean (SD)

^a^P-values from a repeated measures ANCOVA on OGTT Glucose change from pre-ingestion with baseline OGTT glucose (Week 0) as covariate and treatment, insulin use (Yes/No), time, minute (30, 60, 90, 120, 150 and 180), and center as factors and subject as random effect.

### Insulin levels during the OGTT

There were no significant treatment differences in the change from pre-glucose load in insulin levels over the 12-week treatment period (Table 4). Time-dependent changes in insulin levels were observed among the groups. As seen with glucose, mean insulin levels at 30 and 120 minutes post-glucose load were significantly higher in all the treatment groups compared to pre-glucose load levels over the 12-week treatment period. At Week 12, mean insulin levels at 120 minutes post-glucose load were 137.4±179.5, 159.9±165.8, and 123.9±124.6 pmol/L for the placebo, tesamorelin 1 mg and tesamorelin 2 mg groups, respectively.

**Table 4:** Insulin (pmol/L) levels pre-glucose ingestion (0 minute) and changes from pre-glucose ingestion values at 30 and 120 minutes during the OGTT.

	Placebo (n = 16)	Tesamorelin 1 mg (n = 18)	Tesamorelin 2 mg (n = 19)
**Week 0 (Baseline)**			
Pre-glucose ingestion	86.1 (40.9)	168.1 (111.7)	99.5 (67.7)
Change at 30 minutes	115.7 (105.7)	65.4 (78.2)	87.0 (99.30
Change at 120 minutes	140.1 (106.6)	163.3 (199.5)	163.1 (171.8)
**Week 1**			
Pre-glucose ingestion	84.6 (44.7)	159.7 (99.0)	99.3 (52.9)
Change at 30 minutes	119.7 (100.7)	89.9 (118.5)	102.4 (142.0)
Change at 120 minutes	132.5 (122.3)	161.2 (147.7)	162.4 (191.2)
**Week 4**			
Pre-glucose ingestion	83.6 (33.1)	136.4 (73.8)	93.3 (63.0)
Change at 30 minutes	114.1 (84.3)	77.6 (101.2)	98.4 (88.3)
Change at 120 minutes	149.3 (139.7)	158.9 (135.2)	132.7 (126.3)
**Week 8**			
Pre-glucose ingestion	95.4 (41.2)	136.4 (83.5)	108.5 (64.8)
Change at 30 minutes	126.7 (142.0)	119.0 (128.8)	89.0 (97.1)
Change at 120 minutes	102.6 (127.3)	194.2 (158.7)	135.6 (85.8)
**Week 12**			
Pre-glucose ingestion	86.6 (37.1)	154.2 (100.9)	92.2 (44.5)
Change at 30 minutes	85.6 (103.7)	103.4 (124.3)	93.8 (75.3)
Change at 120 minutes	137.4 (179.5)	159.9 (165.8)	123.9 (124.6)
P-value Treatment^a^	0.80		
P-value Week^a^	0.04		
P-value Time Point^a^	<0.001		

Data are reported as mean (SD)

^a^P-values from a repeated measures ANCOVA on OGTT Glucose change from pre-ingestion with baseline OGTT glucose (Week 0) as covariate and treatment, insulin use (Yes/No), week, time point (30, 60, 90, 120, 150 and 180 minutes), and center as factors and subject as random effect.

### Fasting glucose levels

Fasting glucose levels were comparable across treatment groups at baseline. There were no significant differences among groups in fasting glucose over the 12-week treatment period, using the repeated measures ANOVA. Changes from baseline in fasting glucose (mean±SD) were as follows: Week 1: 0.2±1.5, 0.8±2.8, 0.9±2.4 mmol/L; Week 4: 0.4±3.4, 0.2±2.0, 1.0±1.3 mmol/L; Week 8: 0.3±3.1, -0.5±1.4, 0.9±1.2 mmol/L; Week 12: -0.6±2.1, -0.7±1.5, 0.1±2.4 mmol/L, for placebo, tesamorelin 1 mg, and tesamorelin 2 mg, respectively, p = 0.44. There was a borderline effect of time on fasting glucose (p = 0.06), with glucose values higher in the tesamorelin 2 mg group at Weeks 4 and 8 compared to values in the other groups.

### Home glucose measurements

Overall, no significant differences were observed among treatment groups in the change from the lead-in period (baseline) in blood glucose measurements (Table 5). The time of the day had a significant effect on blood glucose measurements. As shown in Table 5, before-lunch glucose levels tended to increase from the lead-in period in the tesamorelin 2 mg group compared to the placebo and tesamorelin 1 mg groups. In addition, time-dependent (Week) changes in blood glucose levels were seen among the groups. The before-lunch changes in glucose levels differed between groups at Weeks 1, 4, and 8. Mean changes from baseline in blood glucose were overall minimal (<1.1 mmol/L, except for the tesamorelin 2 mg before lunch) and were not considered as clinically meaningful.

**Table 5:** Changes from lead-in period (baseline) in mean blood home glucose levels (mmol/L) over the 12-Week treatment period.

	Placebo (N = 16)	Tesamorelin 1mg (N = 18)	Tesamorelin 2mg (N = 19)
**Before Breakfast**			
Lead-in period	7.1 (1.3)	8.2 (1.5)	7.8 (1.7)
Change from Lead-in Period			
Week 1	0.6 (1.3)	0.6 (1.6)	0.6 (1.1)
Week 4	0.4 (1.0)	0.4 (1.8)	1.0 (1.2)
Week 8	0.6 (1.2)	0.3 (1.4)	0.6 (1.3)
Week 12	0.9 (0.9)	1.0 (1.7)	0.7 (1.5)
**Before Lunch**			
Baseline	7.2 (1.5)	8.8 (1.9)	8.4 (1.9)
Change from Lead-in Period			
Week 1	0.0 (1.7)	0.9 (1.9)	1.6 (1.6)
Week 4	0.1 (1.1)	0.4 (2.1)	1.3 (1.4)
Week 8	0.4 (1.0)	0.3 (2.4)	1.0 (1.4)
Week 12	0.6 (1.5)	1.0 (2.6)	0.7 (1.1)
**Before Dinner**			
Baseline	7.1 (1.6)	9.3 (1.7)	8.3 (1.8)
Change from Lead-in Period			
Week 1	0.3 (1.4)	-0.1 (2.4)	1.1 (1.4)
Week 4	0.7 (1.4)	-0.0 (2.1)	0.8 (1.7)
Week 8	0.3 (1.0)	-0.1 (2.9)	0.2 (1.4)
Week 12	0.6 (1.6)	-0.0 (2.4)	0.3 (1.7)
**Bedtime**			
Baseline	9.2 (2.3)	9.8 (1.9)	9.4 (1.6)
Change from Lead-in Period			
Week 1	0.6 (1.7)	0.6 (2.8)	0.8 (2.4)
Week 4	0.4 (2.1)	-0.0 (2.9)	0.3 (1.6)
Week 8	0.1 (2.0)	-0.8 (3.0)	0.8 (1.6)
Week 12	-0.5 (1.6)	0.3 (3.1)	0.8 (2.0)
P-value Treatment^a^	0.77		
P-value Time of the Day^a^	<0.0001		
P-value Week^a^	0.02		

Dare are reported as mean (SD). The mean daily blood glucose level for a time period e.g., lead-in, Week 1, etc…) is defined as the average of the daily results for that time period.

^a^P-values from a repeated measures ANOVA on mean home glucose levels change from baseline with treatment, insulin use (Yes/No), Week (1 to 12), Time of day (Breakfast, Lunch, Dinner and Bedtime), and center as factors and subject as random effect.

### HbA_1c_ levels

The repeated-measures analysis across time did not reveal an overall treatment effect for HbA_1c._ The mean changes (SD) from baseline to Week 12 in HbA_1c_ were -0.5 (0.8), -0.6 (0.8), and 0.1 (0.4) % for the placebo, tesamorelin 1 mg, and tesamorelin 2 mg, respectively (Table 6). The change in HbA_1c_ was statistically significantly different between the placebo and tesamorelin 2 mg groups at Week 12 (p<0.05).

**Table 6:** Changes from baseline in anthropometric and metabolic parameters at Week 12.

	Placebo (N = 16)		Tesamorelin 1 mg (N = 18)		Tesamorelin 2 mg (N = 19)		
	Baseline	Δ	Baseline	Δ	Baseline	Δ	*P-value* ^¶^
Weight (kg)	91±15	-2±3	91±16	-0±2	95±18	-1±3	0.08
BMI (kg/m^2^)	30.3±4.2	-0.7±1.1	32.2±4.2	-0.1 ±0.8	32.1±4.2	-0.4±1.0	0.62
% Body fat	35±7	-0±2	39±7	-1±2	37±7	-1±4	0.96
Waist circumference (cm)	104±12	-2.0±3.8	102±12	-0.3±5.3	106±13	-0.3±5.7	0.29
IGF-I (ng/mL)	131±49	-4±19	102±39	33±37 ^**‡**^	111±31	66±64 ^**§**^	0.02
IGFBP-3 (mg/L)	2.4±0.7	-0.1±0.3	2.3±1.0	0.5±0.7	2.4±0.6	0.4±0.7 ^**§**^	0.12
Fasting glucose (mmol/L)	6.9±2.5	-0.6±2.1	8.7±2.4	-0.7±1.5	7.7±1.4	0.1±2.4	0.44
Relative insulin response (L/pmol)	0.26±0.27	0.02±0.10	0.15±0.27	0.05±0.37	0.07±0.45	0.13±0.56	0.71
HbA_1c_ (%)	6.8±0.9	-0.5±0.8	7.4±0.9	-0.6±0.8	6.9±0.8	0.1±0.4 ^**§**^	0.11
Triglycerides (mmol/L)	2.3±1.3	0.5±1.8	2.3±1.4	-0.3±1.2	2.0±0.9	-0.1±1.1	0.65
Total cholesterol (mmol/L)	4.6±0.8	0.3±0.6	4.6±0.9	0.2±0.4	4.7±1.1	-0.3±0.6 ^**§**^	0.06
HDL cholesterol (mmol/L)	1.1±0.3	0.1.2±0.2	1.2±0.4	0.1±0.1	1.2±0.4	-0.0±0.2	0.56
LDL cholesterol (mmol/L)	2.4±0.7	0.1±0.5	2.5±0.7	0.3±0.5	2.7±0.9	-0.3±0.6	0.15
Non-HDL cholesterol (mmol/L)	3.6±0.9	0.2±0.5	3.5±0.9	0.1±0.4	3.5±1.1	-0.3±0.5 ^**§**^	0.10

Abbreviations: IGF-1, insulin-like growth factor-1; HbA_1c,_ glycated hemoglobin; HDL, high density lipoprotein; LDL, low density lipoprotein.

Data are presented as mean ± SD

^¶^ P-values for the comparison between the changes from baseline in the 3 treatment groups from the repeated-measures ANOVA with main effects for treatment, insulin use (Yes/No), time and center.

^**‡**^ p<0.05 for the comparison between the changes from baseline in the tesamorelin 1 mg group and the placebo group.

^**§**^ p<0.05 for the comparison between the changes from baseline in the tesamorelin 2 mg group and the placebo group.

### Changes in diabetic regimen and diabetes control

During the study, few patients changed medications from the diabetic regimen used at screening and treatment groups were not significantly different in the frequency of changes to diabetic regimens. After 12 weeks of treatment, there was no significant difference among treatment groups in the percentage of patients with investigator-assessed changes in diabetes control. Compared to baseline, diabetes control did not worsen in any patients and was rated better in 6 (55%), 5 (36%), and 7 (47%) patients in the placebo, tesamorelin 1 mg, and tesamorelin 2 mg groups, respectively (p = 0.58). No patients withdrew from the study due to loss of diabetes control.

### Serum lipid parameters

The repeated-measures ANOVA did not reveal any statistically significant differences among the treatment groups in triglycerides, total cholesterol, HDL cholesterol, non–HDL cholesterol, LDL-cholesterol, and triglycerides (Table 6). When compared to baseline, total cholesterol (-0.3±0.6 mmol/L, p<0.05 vs. placebo) and non-HDL cholesterol (-0.3±0.5 mmol/L, p<0.05 vs. placebo) significantly decreased within the tesamorelin 2 mg group at Week 12. HDL cholesterol and triglyceride levels did not significantly change from baseline in any of the treatment groups (Table 6).

### IGF-1 and IGFBP-3 levels

The changes from baseline in IGF-1 were significantly different among the treatment groups Treatment with tesamorelin was associated with a dose-dependent increase in IGF-1 from baseline to Week 12 (mean change from baseline: 33 and 66 ng/mL for the tesamorelin 1 mg and 2 mg groups, respectively, p<0.05 vs. placebo for both groups) (Table 5). IGFBP-3 also significantly increased from baseline in the tesamorelin 2 mg group at Week 12 (mean change from baseline: 0.4 mg/L, p<0.05 vs. placebo) (Table 5).

### Body composition

At Week 12, there were no statistically significant differences among treatment groups in weight, BMI, percent body fat, and waist circumference (Table 5).

### Adverse events

Adverse events were reported in 50% of patients in the placebo group, 39% of patients in the tesamorelin 1 mg, and 58% of patients in the tesamorelin 2 mg group. Adverse events that were reported in more than one patient in the treated groups were nausea (n = 2 in the tesamorelin 2 mg group, 11%), diarrhea (n = 2 in the tesamorelin 1 mg group, 11%), generalized weakness (n = 2 in the tesamorelin 1 mg group, 11%), fatigue (n = 2 in the tesamorelin 2 mg group, 11%), headache (n = 2 in the tesamorelin 2 mg group, 11%), upper respiratory tract infection (n = 2 in the tesamorelin 2 mg group, 11%), and light-headedness (n = 2 in the tesamorelin 1 mg group, 11%). Most adverse events were mild in severity. Anti-tesamorelin antibodies were not detected in any patient after 12 weeks of treatment. During the 4-week follow-up period, 12 AEs were reported in 8 subjects (15%), including 2 subjects in the tesamorelin 1 mg group (cough, dry skin, popular rash) and 3 subjects each in the placebo (anemia, increased serum potassium, upper respiratory tract infection, diaphoresis, pruritus, urinary tract infection) and tesamorelin 2 mg (hypoesthesia, sinusitis, rash) groups. None was reported by more than one subject, and all were mild or moderate in severity. The only AE during the follow-up period that was considered possibly related to study drug was mild hypoesthesia occurring in a subject in the tesamorelin 2 mg group. There were no deaths or serious adverse events during the study or follow-up period.

### Compliance

Mean compliance was 99.5, 98.5 and 97.0% in the placebo, tesamorelin 1 mg and tesamorelin 2 mg groups, respectively.

## Discussion

The use of GH can lead to worsening glucose tolerance in patients with IGT and/or overt diabetes [15]. Most of the GH adverse effects are believed to be linked to supraphysiological increases in GH and IGF-1 levels [16]. Therefore, it has been hypothesized that therapeutic strategies to increase GH and IGF-1 levels within the normal physiological range would result in fewer side effects. Because tesamorelin, a stabilized GHRH analogue, has been previously shown to stimulate GH secretion in a physiological manner [9–10], this study was conducted in patients with type 2 diabetes to assess its potential effects on insulin sensitivity and diabetes control. The results of this study showed that once daily subcutaneous administration of tesamorelin for 12 weeks resulted in a dose-dependent increase in IGF-1, an integrated measure of GH secretion, which remained within the normal reference ranges for the patients’ age groups. More important, the results demonstrate that tesamorelin did not alter the relative insulin response of patients, thus indicating that the drug did not significantly affect glucose tolerance or insulin sensitivity. Our data showing increases from baseline in fasting glucose in the tesamorelin 2 mg group at Weeks 4 and 8, but not at Week 12 suggest a transient increase in fasting glucose following administration of tesamorelin. This finding is in agreement with recent data from Stanley et al. [17] demonstrating initial increases in fasting glucose in HIV-infected patients treated with tesamorelin, which were reversed after 6 months of treatment, with glucose returning to baseline values. Home blood glucose levels increased before lunch in the tesamorelin 2 mg group at Weeks 1, 4, and 8. However, the increases were mild and of no clinical significance. Our data indicate that HbA_1c_ slightly increased from baseline in the tesamorelin 2 mg group at Week 12. It is possible that the slight increase in HbA_1c_ seen at Week 12 in the 2 mg tesamorelin group could reflect the increases in fasting glucose observed at Weeks 4 and 8. Alternative reasons for the observed increase in HbA_1c_ in the 2 mg tesamorelin group could be the relatively small study’s sample size or changes in pharmacotherapy. Of note, control of diabetes, as assessed by the investigator, was improved at the end of the study in approximatively 50% of patients receiving tesamorelin 2 mg. A recent study showed that administration of tesamorelin to HIV-infected patients with excess abdominal fat for 52 weeks did not affect glucose parameters, including fasting glucose, 2-hour glucose on OGTT, fasting insulin, and HbA_1c_ [18]. Nonetheless, because GH may induce glucose intolerance in some individuals, patients receiving ongoing treatment with tesamorelin should be monitored periodically for potential changes in glucose metabolism [18].

Overall, our findings are in agreement with previous studies demonstrating that GHRH analogues restore physiologic GH and IGF-1 levels without inducing insulin resistance and/or hyperglycemia in various populations, including healthy middle-aged and elderly subjects [5, 7, 8], GH-deficient children [19], and patients with HIV-associated lipodystrophy [6]. In contrast, chronic administration of pharmacological or low doses of rhGH has been shown to negatively affect glucose metabolism in GH-deficient children and adults [1, 20, 21], abdominally obese subjects [22–23], and HIV-infected patients with wasting [24] or lipodystrophy [25–26].

Several studies have been conducted to investigate the mechanism underlying the induction of insulin resistance by pharmacological doses of GH. Free fatty acids derived from GH-stimulated lipolysis have been proposed to contribute to the diabetogenic properties of GH through a mechanism involving an increase in hepatic glucose output and a decrease in peripheral glucose oxidation according to the glucose-fatty acid cycle utilization, i.e. a switch from using glucose to lipids [27]. Supporting this possibility are the results from Bramnert et al. [28] demonstrating a close correlation between serum free fatty acids and the rate of lipid oxidation in GH-deficient patients. The molecular mechanism by which chronic GH excess affects glucose metabolism is not well defined. The results from several *in vitro* and *in vivo* studies suggest that long-term exposure to GH may induce insulin resistance by several mechanisms, including increased expression of cellular proteins that inhibit the insulin receptor signaling [29–30], a decrease in the expression of several key proteins involved in carbohydrate metabolism, as well as a decrease in the insulin-mediated activation of glycogen synthase in the liver and muscle [31]. It is believed that the beneficial effect of GHRH analogues on glucose metabolism is due to the fact that feedback inhibition of IGF-1 on GH secretion by the pituitary remains intact, thus preventing adverse effects due to GH excess [11, 32, 33]. In agreement with this possibility, our data show that mean IGF-1 levels remained within the normal range following administration of tesamorelin to type 2 diabetic patients.

Treatment with tesamorelin for 12 weeks was associated with a significant decrease from baseline in LDL cholesterol and non-HDL cholesterol, whereas other lipid parameters remained unchanged. Our results are consistent with other studies in humans, which demonstrated a significant reduction in plasma cholesterol after GH replacement therapy [3, 34]. The available evidence indicates that GH may reduce plasma cholesterol levels by stimulating hepatic LDL receptor expression [35–36] and/or by increasing the activity of acyl coenzyme A cholesterol acyltransferase (ACAT), the hepatic rate-limiting enzyme in bile acid biosynthesis [37]. Our data are at variance with previous results showing an increase in serum triglyceride levels after short-term administration of rhGH [38–39]. This increase may be explained by both an increased flux of free fatty acids to the liver and a direct stimulatory effect on the esterification of oleic acid into triglycerides and phospholipids in hepatocytes [40].

This study has some limitations. First, the sample size was relatively small; hence, the results should be interpreted with caution. Further studies with a larger sample size are needed to validate the findings of the current study. Second, although the duration of 12 weeks of treatment was adequate to discern important changes in glycosylated hemoglobin (HbA_1c_), a study of longer duration is necessary to gather important information about the long-term safety of tesamorelin in patients with type 2 diabetes. Third, the study population consisted largely of Caucasians, which may limit the generalization of the study. Fourth, potential changes in diet and exercise were not objectively measured in the study subjects. Fifth, it is possible that the adverse effects of tesamorelin on glucose tolerance may have been masked due to the concomitant use of oral hypoglycemic agents. In addition, no correction was made for multiple outcomes or multiple comparisons.

Overall, our data indicate that daily administration of tesamorelin for 12 consecutive weeks to type 2 diabetic patients was not associated with significant changes in relative insulin response and/or diabetes control. In addition, treatment with tesamorelin was associated with improvements in lipid parameters.

## Supporting information

S1 CONSORT checklist (DOCX)

S1 Protocol (PDF)
