# Supplementary material for: Safety and metabolic effects of tesamorelin, a growth hormone-releasing factor analogue, in patients with type 2 diabetes: A randomized, placebo-controlled trial
Source: PLoS One. 2017 Jun 15;12(6):e0179538. doi: 10.1371/journal.pone.0179538 (PMC5472315; doi:10.1371/journal.pone.0179538)
Supplement: S1 Protocol — (PDF) [file pone.0179538.s002.pdf]

Clinical Development

TH9507

Clinical Study Protocol

TH9507/II/DIABETIC/006

A Double-Blind, Randomized, Parallel, Placebo-Controlled 12-Week Evaluation of the Safety of Two Doses of TH9507 in Subjects with Stable, Type 2 Diabetes Mellitus

Development Phase: II

Release date: 31 August 2001
Amendment 1: 20 November 2001

This document contains confidential information belonging to Theratechnologies Inc. Except as may be otherwise agreed to in writing, by accepting or reviewing this document, you agree to hold such information in confidence and not to disclose it to others (except where required by applicable law) nor use it for unauthorized purposes. In the event of actual or suspected breach of this obligation, Theratechnologies should be promptly notified.

Signature page for Investigators

Compound name / number: TH9507

Protocol Number: TH9507/II/DIABETIC/006

Protocol Title: A Double-Blind, Randomized, Parallel, Placebo-Controlled 12-Week Evaluation of the Safety of Two Doses of TH9507 in Subjects with Stable, Type 2 Diabetes Mellitus

I have read this protocol and agree to conduct this trial in accordance with all stipulations of the protocol and in accordance with ICH/GCP guidelines and the Declaration of Helsinki.

______________________________	______________________________
Name of investigator(s)	Date
______________________________	______________________________
Name of co-investigator(s)	Date

1. Introduction

The somatotropic axis regulates many essential physiologic functions. Although growth hormone (GH) was originally recognized for its primary role in effecting growth in stature, its importance in preserving anabolism and its involvement in tissue repair during adulthood have recently been more fully documented. The aging process, however, is associated with a blunting of basal and stimulated GH secretion[1-8]. This early and progressive decrease in spontaneous GH secretion is associated with a decrease in blood levels of insulin-like growth factor 1 (IGF-1), the principal effector and marker of GH anabolic actions[9-11].

Experimental attempts to restore complete functionality of the somatotropic axis in aged patients have included the administration of exogenous recombinant human growth hormone (rhGH) and analogues of growth hormone releasing factor (GRF). Administration of rhGH to these patients, however, has been associated with a variety of adverse effects including hyperglycemia, insulin resistance, fluid retention, carpal tunnel syndrome, and gynecomastia [12-15]. These adverse effects have been shown to be more frequent in patients with the highest IGF-1 levels[15]. Importantly, the IGF-1 negative feedback on GH secretion is inoperative during treatment with exogenous rhGH, and exogenous rhGH is unable to mimic the physiologic, pulsatile rate of endogenous GH secretion[16]. While GRF is able to preserve the physiologic pulsatile pattern of GH secretion, rapid hydrolysis of natural GRF by dipeptidyl-amino peptidase in serum limits its efficacy as a treatment modality. Administration of a GRF analogue has been associated with enhanced insulin sensitivity in males [17], in contrast to the insulin resistance observed with the administration of rhGH.

TH9507, a GRF analogue being developed by Theratechnologies Inc., includes the natural 44 amino acid sequence of human GRF (hGRF), on which a hexenoyl moiety (a C6 chain with a double bond on position 3) has been anchored on Tyr 1 at the N-terminus part of the molecule. The presence of this hydrophobic side chain is believed to provide steric hindrance that prevents or delays the coupling of dipeptidyl-amino peptidase to TH9507, resulting in enhanced stability in serum. In fact, the plasma half-life of TH9507 was estimated to be 8 hours (*in vitro*) in comparison to hGRF, which was degraded in minutes[18]. In a Phase 1b study TH9507 increased IGF-1 levels in healthy older men to levels typically observed in young adults[19]. Since TH9507 has the natural sequence of GRF and is relatively resistant to degradation by peptidases in serum, it combines three key features for optimal efficacy and safety in aged patients: specificity on GH secretion; induction of GH secretion in a pulsatile, physiologic manner; and a powerful effect on IGF-1 secretion. These characteristics are expected to translate into clear clinical benefits in a number of targeted therapeutic indications.

1.1 Rationale

The clinical development program for TH9507 includes its use in patients who could benefit from enhancement of anabolic effects, including patients with COPD, hip fractures, and other age-related disorders associated with somatopause. As noted, different changes in insulin sensitivity have been observed with rhGH and GRF analogue administration. It is anticipated that a number of patients to be enrolled in future trials of TH9507 will have glucose intolerance and/or overt diabetes. Therefore, the purpose of this study is to investigate the safety of daily administration of TH9507 in patients with type 2 diabetes mellitus, in particular, the potential for effects on insulin sensitivity and diabetic control.

2. Study Objectives

The primary objective will be to evaluate the safety of two doses of TH9507 administered over 12 weeks by daily subcutaneous injections in patients with stable type 2 diabetes.

3. Investigational Plan

3.1 Overall Study Design

This is a randomized, double-blind, placebo-controlled, parallel group, multicenter study of two doses of TH9507 (1 mg and 2 mg) administered daily by subcutaneous injection for 12 weeks.

After screening and a 14-day lead-in period, subjects will be randomized by insulin use (see Section 4.1.6) to one of three treatment groups. Subjects will be evaluated after 1, 4, 8, and 12 weeks of treatment and at a follow-up visit at Week 16.

The study schematic is presented in Figure 3.1-1.

Figure 3.1-1 Study Schematic

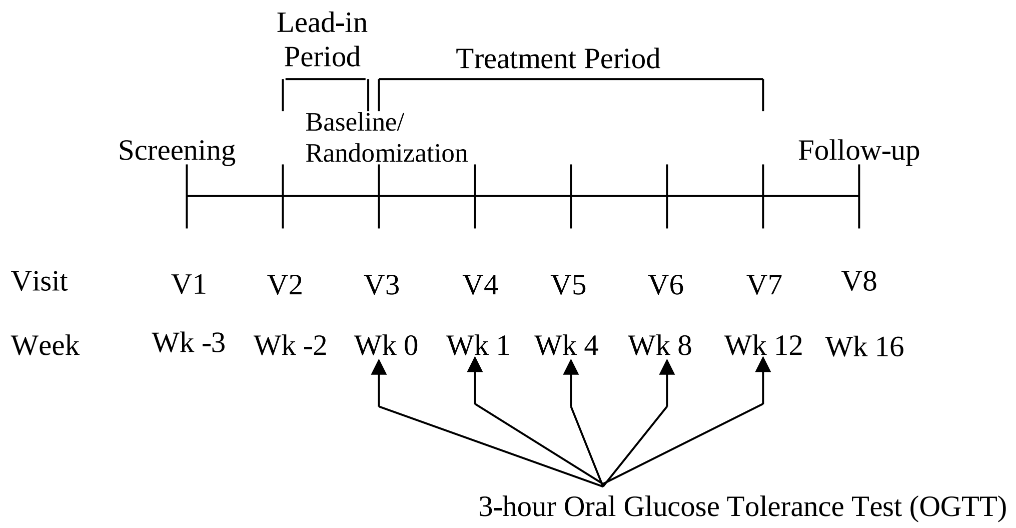

3.2 Discussion of Design

This study will incorporate a two-week lead-in period prior to randomization for the purpose of establishing and characterizing daily dietary patterns and daily blood glucose measurements prior to the randomization visit. Further, the lead-in period will provide patients with the opportunity to become accustomed to required study procedures such as the completion of a diary to monitor diet, exercise, blood glucose, and hypoglycemic episodes. The duration of 12 weeks of treatment is adequate to discern important changes in glycosylated hemoglobin (HbA_{1c}).

3.3 Study Population

3.3.1 Patient Population

A total of approximately 45 patients with stable type 2 diabetes on stable diabetes treatment regimen (receiving oral hypoglycemics with or without insulin) will be enrolled into the study. Fifteen (15) subjects will be randomized to each treatment group to ensure that approximately 13 subjects per group complete the study. After 33 patients have been randomized based on insulin use (insulin use or non-insulin use), enrollment may be halted for that group but continued in the other group.

3.3.2 Inclusion and Exclusion Criteria

Inclusion criteria

1. Male or postmenopausal or surgically sterilized female subjects, of 50 years of age or older;
2. Documented diagnosis of type 2 diabetes as defined by the American Diabetes Association[20];
3. Diagnosis of type 2 diabetes for at least 3 months prior to screening;
4. Subjects on stable diabetes treatment regimen (receiving oral hypoglycemics with or without insulin) for at least 2 months prior to screening;
5. Screening and pre-randomization HbA_{1c} <10.0%, according to central laboratory;
6. Subjects with a Body Mass Index between 25 and 38 kg/m^2.
   (BMI = Weight [kg] ÷ {Height [m]}^{2});
7. Subjects willing to perform specified home blood glucose monitoring and comply with all study protocol requirements;
8. Signed informed consent.

Exclusion criteria

1. Serum creatinine >2 mg/dL;
2. Fasting triglyceride >1000 mg/dL;
3. Albuminuria >200 mcg/24 hours (this assessment may be obtained during the lead-in period);
4. Positive mammography (if female) or prostate-specific antigen (PSA) or prostate examination for cancer(if male);
5. Donation of blood during the 30 days prior to the screening visit. Donation of blood is also prohibited during the study;
6. Use of oral or parenteral glucocorticoids in the 30 days prior to screening;
7. Use of any experimental or marketed growth hormone (GH), GH secretagogues (e.g., growth hormone releasing factor (GRF) products), IGF-1, or IGFBP-3 during the previous 6 months;
8. Subjects with two or more severe hypoglycemia episodes within the past 6 months, or any hospitalization or emergency room visit due to poor glycemic control within the past 6 months. Similarly, during the lead-in period, any subject with more than one severe hypoglycemic episode or any hospitalization or emergency room visit due to poor glycemic control will be excluded from randomization;
9. History of or presence of active concomitant conditions or diseases (e.g., myocardial infarction, poorly controlled hypertension, thyroid disease, rheumatoid arthritis, seizure disorder, diabetic neuropathy, diabetic retinopathy [except patients

with only microaneurysms on fundus examination]) that would interfere with the protocol conduct and endpoint measurements;

10. Subjects with a major surgical operation during the 30 days prior to screening;
11. Subjects with known hypopituitarism, history of pituitary tumor/surgery, head irradiation, or severe head trauma.
12. Current cancer or history of cancer, except non-melanomatous skin cancer;
13. Subjects with active infection at any body site or a history of severe infection (requiring oral or parenteral treatment) during the 30 days prior to screening;
14. Subjects with drug or alcohol dependence or abuse;
15. Subjects with clinically significant abnormalities on screening laboratory evaluation (unless discussed with and approved by the medical monitor);
16. Subjects with allergy to synthetic growth hormone products or their excipients;
17. Subjects who have previously received growth hormones in any clinical trial; and
18. Participation in a trial of an experimental drug or device within 90 days prior to screening.

3.3.3 Interruption or Discontinuation of Treatment

Before interrupting or discontinuing study drug, the investigator must contact the sponsor or designee (except in the case of an emergency) to discuss the proposed reason. It will be documented whether or not each subject completed the clinical study. If any subject's study treatment or observations were discontinued, the reason will be recorded. Reasons that a subject may discontinue participation in this clinical study may be one or more of the following:

- Adverse event(s)
- Abnormal laboratory value(s)

  Hemoglobin: decrease below 9.0 gm/dL
  AST: ≥3 X ULN (upper limit of normal)
  ALT: ≥3 X ULN
  LDH: ≥3 X ULN
  Alkaline Phosphatase: ≥2 X ULN
  Triglyceride: ≥1000 mg/L
  Creatinine: >2 mg/dL

- **Any fasting glucose >300 mg/dL and/or any HbA_{1c} value of >12% in spite of ad lib adjustments to oral hypoglycemic agents and/or insulin dosage.**
- Subject withdrew consent
- Lack of compliance to protocol requirements and/or procedures
- Lost to follow-up

- Administrative deviations which preclude satisfactory completion of protocol assessments

3.4. Treatment

3.4.1 Investigational Drug and Reference Therapy

Active drug and placebo will be provided as lyophilized powder in vials of identical appearance and supplied in blister cards at each monthly visit. The active drug is supplied as 1.1 mg of TH9507 (peptide content) with 55 mg of mannitol. The placebo is composed of 55 mg of mannitol. The study drugs will be supplied as follows:

**Dose**	**No. of Vial of Active Drug (A)**	**No. of Vial of Placebo (B)**
1 mg	1 vial of 1.1 mg active drug	1 vial of mannitol
2 mg	2 vials of 1.1 mg active drug	-
Placebo	-	2 vials of mannitol

Each vial will be reconstituted with 1.1 mL of sterile water for injection (provided by the sponsor). One mL of each preparation is then taken out of each vial using a 3-mL syringe. The total volume will be 2 mL. The preparation is then injected subcutaneously.

The lyophilized vials will be kept at -20°C at the investigational sites. The supplies of study drug dispensed to the subjects will be kept refrigerated between 2ºC and 8ºC at the subject's home for daily reconstitution and use.

Subjects will self-inject 2 mL of study drug subcutaneously (using 1 mL each from vial A and vial B) at rotating sites every morning at 9:00AM ± 3 hour, (i.e., between 6AM and noon). The subject may elect to have a family member or a health professional administer the study drug. The study drug may be administered in either one or two subcutaneous injections at each dosing time at one or two sites. The study drug will be administered at the General Clinical Research Centers (GCRCs) at Visits 3, 4, 5, and 6.

Medication labels will comply with the regulatory requirements.

3.4.2 Treatment Assignment

Patients will be assigned to treatment groups in a randomized fashion. Drugs will be numbered in such a way that patients at each investigational site will receive the treatment sequentially as they qualified to be randomized at Visit 3. The patient randomization number will be the lowest available treatment number.

3.4.3 Blinding

There will be three sets of sealed envelopes containing the randomization codes that will be generated by an external consultant. One set will be provided to the packaging supplier, another will be provided to the investigator, and another will be kept at PharmaResearch Corporation.

The randomized code will be broken ONLY in case of an emergency. If possible, the investigator must contact the Sponsor or designee before unblinding the code to discuss the event leading to potential unblinding such as a possibly drug-related serious adverse event.

Randomization data are kept strictly confidential, accessible only to authorized persons, until the time of unblinding. At the conclusion of the trial, the occurrence of any emergency code breaks will be verified after return of all code break reports and unused drug supplies to Theratechnologies. Only when the study has been completed, the data file verified, and the protocol violations determined, will the drug codes be broken and made available for data analysis.

[For details of the emergency procedure for unblinding of individual patients in cases of emergency see section 6.2.]

3.4.4 Concomitant Therapy

Oral or parenteral glucocorticoids will not be permitted during the study. Hormone replacement therapy for female subjects will be allowed. All other concomitant drugs should be maintained at constant dose during the study, if possible. Concomitant drugs will be recorded in the Case Report Forms.

Dose adjustments for insulin and/or oral hypoglycemics during the conduct of the trial will be at the discretion of the investigators.

3.4.5 Treatment Compliance

Compliance will be checked by counting the number of empty vials returned at each visit.

3.5 Visit Schedule and Assessments

The schedule of assessments is presented in Appendix 1.

3.5.1 Visit Schedule

Visits 1 and 2 are conducted prior to the baseline/randomization Visit 3, which is designated as Week 0.

VISIT 1: (Week –3, Screening)

- Obtain signed informed consent.
- Review inclusion and exclusion criteria.
- Obtain medical history.
- Review concomitant medications.
- Perform physical/neurological examinations (including screening for diabetic sensory neuropathy).
- Obtain 12-lead electrocardiogram.
- Obtain Vital signs (blood pressure, respiratory rate, pulse rate).
- Obtain body weight.
- Calculate body mass index.

- Perform funduscopy with dilation by optometrist or ophthalmologist (may be performed during the lead-in period) and examine for evidence of diabetic retinopathy (microaneurysms will not exclude the patient from participating in the study).
- Obtain blood for clinical laboratory screening tests (hematology, blood chemistry, lipid profile, and urinalysis-subjects with minimal proteinuria [<2+] may continue with screening).
- Obtain blood for glycosylated hemoglobin (HbA_{1c}) and insulin antibodies.
- Schedule mammography (for females) and prostate examination/PSA (for males) if not done within six months of screening.
- Collect 24-hour urine for albumin (may be performed during the lead-in period).
- Schedule Visit 2 to occur 6-8 days following Visit 1.

VISIT 2: (Week –2 ± 1 day, Start of lead-in period)

- Review inclusion and exclusion criteria.
- Assess adverse events.
- Review concomitant medications.
- Obtain body weight.
- Obtain vital signs (BP, RR, PR).
- Dispense diary (to monitor home blood glucose measurement, hypoglycemic episodes, study drug administration**, adjustments in oral hypoglycemic agents and/or insulin dosage**, diet, and exercise) and provide instructions regarding completion of the diary.
- Dispense home glucose meter with instructions.
- Dispense boxes of strips and lancets.
- Instruct the subject to withhold oral sulfonylurea, insulin, and other insulin secretagogues but he/she may take oral non-sulfonylurea hypoglycemic medication (if oral insulin sensitizer) in the morning of Visit 3.
- Instruct the subject to collect urine the day before the scheduled visit (starting at 6AM) until the morning (6AM) of the clinic visit.
- Dietician interview and assessment – caloric intake assessment, skin thickness (Harpenden), waist/hip measurements, and body fat mass (Lange).
- Schedule Visit 3 to occur 12-16 days after Visit 2.

VISIT 3: (Week 0 ± 2 days, Baseline and Randomization)

- Review inclusion/exclusion criteria.
- Assess adverse events.
- Review concomitant medications.
- Obtain body weight.

- Obtain vital signs (BP, RR, PR).
- Perform physical/neurological examinations.
- Review/dispense diary. Subjects should be at least 90% compliant with the diary and diet in order to continue participation in the study.
- Replenish boxes of strips and lancets, if necessary
- Download home glucose readings from 14-day lead-in period.
- Instruct the subjects to call the clinic for any home blood glucose value that is >250 mg/dL.
- Dietician interview and assessment – caloric intake assessment, skin thickness (Harpenden), waist/hip measurements, and body fat mass (Lange).
- Collect 24-hour urine for creatinine and nitrogen.
- Obtain blood (fasting) for laboratory tests – IGF-1, IGFBP-1, IGFBP-3, sera for antibodies to insulin and TH9507, lipid profile, hematology, chemistry, HbA_{1C}
- Perform oral 3-hr GTT with 75Gm Glucose (Glucola).
- Obtain blood for insulin and glucose levels every 30 minutes during OGTT.
- Assign randomized study treatment to subject.
- Administer first dose of blinded study drug in clinic at approximately noon.
- Dispense study drug supply for one week (8 doses).
- Instruct the subject to collect urine the day before the scheduled visit (starting at 6AM) until the morning (6AM) of the clinic visit.
- Schedule Visit 4 to occur 6-8 days following Visit 3.

VISIT 4: (Week 1 ± 1 day; Treatment Period)

- Assess adverse events.
- Review concomitant medications.
- Return all used and unused study drugs.
- Obtain body weight.
- Obtain vital signs (BP, RR, PR).
- Review/dispense diary.
- Download home glucose readings from previous 6-8 days.
- Replenish boxes of strips and lancets, if necessary.
- Collect 24-hour urine for creatinine and nitrogen.
- Obtain blood (fasting) for laboratory tests – IGF-1, IGFBP-1, IGFBP-3, lipid profile, hematology, chemistry.
- Perform oral 3-hr GTT with 75Gm Glucose (Glucola).
- Obtain blood for insulin and glucose levels every 30 minutes during OGTT.
- Administer blinded study drug while in clinic at approximately noon.
- Dispense study drug supply for three (3) weeks (24 doses).

- **Remind the subjects to call the clinic for any home blood glucose value that is >250 mg/dL**.
- Instruct the subject to withhold oral sulfonylurea, insulin, and other insulin secretagogues but he/she may take oral non-sulfonylurea hypoglycemic medication (if oral insulin sensitizer) in the morning of Visit 4.
- Instruct the subject to collect urine the day before the scheduled visit (starting at 6AM) until the morning (6AM) of the clinic visit.
- Schedule Visit 5 to occur 19-23 days after Visit 4.

VISIT 5 and VISIT 6: (Week 4 ± 2 days and Week 8 ± 3 days, Treatment period)

- Assess adverse events.
- Review concomitant medications.
- Return all used and unused study drugs.
- Obtain body weight.
- Obtain vital signs (BP, RR, PR).
- Dietician interview and assessment – caloric intake assessment, skin thickness (Harpenden) , body fat mass (Lange); and waist/hip measurements.
- Review/dispense diary.
- Download home glucose readings for the period since the previous visit.
- Collect 24-hour urine for creatinine and nitrogen.
- Obtain blood for laboratory tests – IGF-1, IGFBP-1, IGFBP-3, lipid profile, hematology, chemistry, HbA_{1C}.
- Perform oral 3-hr GTT with 75Gm Glucose (Glucola).
- Obtain blood for insulin and glucose levels every 30 minutes during OGTT.
- Administer daily dose of blinded study drug in clinic at approximately noon.
- Dispense study drug supply sufficient for four weeks (32 doses).
- Replenish boxes of strips and lancets, if necessary
- **Remind the subjects to call the clinic for any home blood glucose value that is >250 mg/dL**.
- Instruct the subject to withhold oral sulfonylurea, insulin, and other insulin secretagogues but he/she may take oral non-sulfonylurea hypoglycemic medication (if oral insulin sensitizer) in the morning of Visit 6 and Visit 7.
- Instruct the subject to collect urine the day before the scheduled visit (starting at 6AM) until the morning (6AM) of the clinic visit.
- Schedule Visit 6 to occur 25-31 days following Visit 5, OR
- Schedule Visit 7 to occur 25-31 days following Visit 6.

VISIT 7: (Week 12 ± 3 days)

- Assess adverse events.
- Review concomitant medications.

- Return all used and unused study drugs.
- Obtain body weight.
- Obtain vital signs (BP, RR, PR).
- Determine body mass index.
- Perform funduscopy with dilation by optometrist or ophthalmologist (may be performed within 5 days of date of Visit 7).
- Perform physical/neurological examinations (including examination for diabetic sensory neuropathy).
- Review/dispense diary.
- Download home glucose readings from previous 4 weeks.
- Dietician interview and assessment – caloric intake assessment, skin thickness (Harpenden), waist/hip measurements, and body fat mass (Lange).
- Collect 24-hour urine for creatinine and nitrogen.
- Obtain blood (fasting) for laboratory tests – IGF-1, IGFBP-1, IGFBP-3, serum for TH9507 antibody, lipid profile, hematology, chemistry, HbA_{1C}.
- Perform oral 3-hr GTT with 75Gm Glucose (Glucola).
- Obtain blood for insulin and glucose levels every 30 minutes during OGTT
- **Remind the subjects to call the clinic for any home blood glucose value that is >250 mg/dL.**
- **Investigator completes global assessment of diabetes control**
- Replenish boxes of strips and lancets, if necessary
- Subject receives NO STUDY DRUG AT THIS VISIT.
- Schedule Visit 8 (Post-treatment Visit) to occur 25-31 days following Visit 7.

VISIT 8: (Week 16 ± 3 days; Follow-up Visit; 16 weeks after randomization; 4 weeks post-treatment)

- Assess adverse events.
- Review concomitant medications
- Obtain body weight.
- Obtain vital signs (BP, RR, PR).
- Obtain blood for laboratory tests (hematology; blood chemistry; lipid profile)
- Collect urine for urinalysis.
- Schedule mammography (for females) and prostate examination/PSA (for males).
- Dietician interview and assessment – caloric intake assessment, skin thickness (Harpenden) , body fat mass (Lange), and waist/hip measurements.
- Review diary.
- Download home glucose readings for the period since Visit 7.

3.5.2 Safety Assessments

This is a study to assess the safety of 12 weeks of daily administration of TH9507. Safety assessments will include the monitoring and recording of all adverse events and any serious adverse events; the sequential assessment of hematology, blood chemistry, and urinalysis parameters; the monthly monitoring of HbA_{1c}, periodic assessments of lipid profiles, including HDL, LDL, total cholesterol, and triglycerides; monitoring for antibodies to TH9507 before and after 12 weeks of treatment; and periodic measurements of blood pressure, as well as performance of physical examinations including screening testing for diabetic sensory neuropathy. This study will seek to assess any clinically meaningful changes in insulin sensitivity through use of sequential periodic oral glucose tolerance tests with paired insulin and glucose levels to determine insulin:glucose ratios.

3.5.2.1 Adverse Events

Information regarding adverse events will be collected and recorded on the Adverse Events case report form. An adverse event is any undesirable sign symptom or medical condition occurring after the subject is enrolled in the study, whether considered drug-related or not. Medical conditions/diseases present before starting study drug are only considered adverse events if they worsened after starting study drug. Study drug includes any drug(s) under evaluation in the study, including reference drug(s), placebo, or any other drug required by the protocol.

Adverse events, whether volunteered by the subject, discovered during general questioning by the investigator, or detected through physical examination, laboratory tests or other means will be recorded on the Adverse Event case report form and followed carefully until they resolve. Abnormal laboratory values or test results should not generally be considered adverse events, unless they induce clinical signs or symptoms or require therapeutic intervention, when they should be recorded on the Adverse Events case report form using an appropriate diagnostic description.

As far as possible, each adverse event will also be described by:

- its duration (start and end dates),
- the severity grade (mild, moderate, severe),
- its relationship to the study drug (suspected / not suspected),
- the action(s) taken

Examples of the severity grade, relationship to study drug and actions taken, as presented in the case report form are provided in **section 6.3: Instruction for completing Adverse Event case report forms.**

3.5.2.2 Serious adverse events

Information regarding serious adverse events will be collected and recorded on the Serious Adverse Event (SAE) Report Form. A serious adverse event is defined as any untoward medical occurrence that occurs at any dose and:

- results in death,
- is life-threatening,
- requires inpatient hospitalization or prolongation of existing hospitalization,

- results in persistent or significant disability / incapacity, or
- is a congenital anomaly/birth defect in the offspring of a subject who received study drug.

Important medical events that may not be immediately life-threatening or result in death or hospitalization may be considered an SAE when, based upon appropriate medical judgement, they may jeopardize the subject or may require medical or surgical intervention to prevent one of the outcomes listed in the definition above. Examples of such medical events include allergic bronchospasm requiring intensive treatment in an emergency room or at home, blood dyscrasias, or convulsions that do not result in inpatient hospitalization, or the development of drug dependency or drug abuse.

Any serious adverse event occurring after a subject has signed the informed consent form and until four (4) weeks after the stopping study drug must be reported by the investigator to Theratechnologies or designee within 24 hours of learning of its occurrence, even if not seeming to be drug-related. Follow-up information about a previously reported serious adverse event must also be reported to Theratechnologies or designee within 24 hours of receiving it. The investigator should send the completed Serious Adverse Event Report Form by fax to the local representative responsible (**see Section 6.1: Instructions for rapid notification of serious adverse events**).

3.5.2.3 Laboratory Evaluations

Approved laboratories will be used for all assays of TH9507 antibody, IGF-1, IGFBP-1, IGFBP-3, glucose, insulin, insulin antibody, hematology analytes, blood chemistry analytes, and urinalysis. Names, addresses and information concerning sample collections and shipment will be provided separately.

3.5.2.3.1 Hematology

Hemoglobin, hematocrit, platelet count, WBC count, RBC count, and differential.

3.5.2.3.2 Blood Chemistry

AST, ALT, alkaline phosphatase, total bilirubin, creatine phophokinase (CPK), lactate dehydrogenase (LDH), creatinine, urea, amylase, lipase, uric acid, glucose, cholesterol, LDL, HDL, triglycerides, sodium, potassium, calcium, chloride, protein, albumin.

3.5.2.3.3 Urinalysis

Specific gravity, pH, leukocytes, protein, glucose, ketones, bilirubin, RBCs, nitrites, urobilinogen.

3.5.2.3.4 24-Hour Quantitative Urine Tests

Albumin, creatinine, nitrogen, and total urine volume

3.5.2.3.5 Special Tests

HbA_{1c}, insulin and TH9507 antibodies

3.5.2.3.6 Oral Glucose Tolerance Test (OGTT) with paired glucose and insulin levels

Blood glucose and insulin will be measured at 0 minutes (prior to ingestion of glucose), 30 minutes, 1, 1.5, 2, 2.5, and 3 hours. For these determinations, oral sulfonylureas, insulin, and other insulin secretagogues must be withheld on the morning of the OGTT. Insulin sensitizers need not be withheld at such times.

3.5.2.3.7 Home Blood Glucose

Home blood glucose will be obtained 4 times per day (before breakfast, lunch, and dinner, and at bedtime) using a standard blood glucose meter. The glucose values will be downloaded into a computerized recall system. Subjects will be instructed to record the glucose values in their diaries **and to call the clinic for any glucose value that is >250 mg/dL.**. Subjects will be instructed to maintain a consistent meal schedule throughout the study**.**

3.5.2.4 Vital Signs

Respiration rate, pulse rate and sitting blood pressure after 5 minutes of rest.

3.5.2.5 Physical examination

Information about the physical examination must be present in the source documentation at the study site. Significant findings that are present prior to the start of the study drug must be included in the Relevant Medical History case report from or Current Medical Conditions case report form. Significant findings made after the start of the study drug which meet the definition of an AE must be recorded in the Adverse Event case report form.

The examination of the following will be performed: eyes, ears, nose and throat; heart; peripheral vasculature; lungs; muskuloskeletal system, abdomen, neurologic function (with testing for diabetic sensory neuropathy), endocrine system, skin and lymph nodes.

3.5.2.6 Body Fat Mass

Body fat mass will be estimated from measurements with Lange skin-fold calipers at biceps, triceps, subscapular, and iliac crest sites.

3.5.2.7 Skin Thickness

Skin thickness will be measured at four sites (the right and left hand and the right and left volar forearm) using Harpenden calipers.

3.5.2.8 Body Weight

Subjects will be weighed clothed without shoes.

3.5.2.9 Waist/Hip Measurements

Waist will be measured at the smallest abdominal circumference while hips will be measured at the largest gluteal circumference using a tape measure.

3.5.3 Drug Concentration and Pharmacokinetic Evaluations

Not applicable.

4. Statistical Methods

4.1 Statistical Methods to be Employed

4.1.1 Study Objectives

This is a Phase II, placebo-controlled, comparative study. The primary objective is to evaluate the safety of two doses (1 mg and 2 mg by daily subcutaneous injection) of TH9507 relative to placebo administration, over a 12-week period in patients with stable, type 2 diabetes.

The primary endpoint is the change in relative insulin response following the oral ingestion of glucose over the 12-week treatment period. The relative insulin response is defined as the ratio of the incremental plasma insulin response above basal at 30 minutes to that of plasma glucose, divided by basal plasma insulin levels[21].

The secondary objectives are to evaluate the effects of 12 weeks of daily treatment with TH9507 on HbA_{1c}, blood glucose measurements, **use of oral hypoglycemic agents and/or insulin and control of diabetes.**

The secondary endpoints include the change in HbA_{1c} at Week 12 compared to baseline and changes in mean daily serum glucose concentrations compared to the 14 day lead-in period values**, changes in the number of dose adjustments per week for insulin and/or oral hypoglycemic agents compared to the 14 day lead-in period, and the number of subjects with a change in the control of diabetes.**

4.1.2 Study Population

The sample in this study is to be drawn from a target population of stable, type 2 diabetic patients. Satisfying the inclusion and exclusion criteria, these patients will constitute a recognizable and identifiable group as they will specifically

- Have a documented diagnosis of type 2 diabetes mellitus according to the American Diabetes Association,
- Demonstrate stable type 2 diabetes mellitus for at least 3 months prior to study entry,
- On stable diabetes treatment regimen (oral hypoglycemics with or without insulin) for at least 2 months prior to screening,
- Have screening $HbA_{1c} < 10.0\%$,
- Have a Body Mass Index between 25 and 38 add kg/m^2, and
- Will not have used oral or parenteral glucocorticoids for at least 30 days prior to screening.

4.1.3 Statistical Model/Design

The study design is that of a multicenter, double-blind, placebo-controlled, parallel group evaluation of the effects of two doses of TH9507 over 12 weeks. Serial determinations will be

assessed using a repeated measures parallel group design.

4.1.4 Null and Alternative Hypotheses

The null hypothesis is that each active dose does not result in a change in the relative insulin response over the 12-week treatment period. The alternative hypothesis is that at least one of the two doses of TH9507 or placebo produces a change in the primary endpoint over the 12-week treatment period. Thus the primary analysis is two-sided since the direction of the putative difference is not specified.

Assessment of these hypotheses may be conducted by comparing the changes from baseline at Week 12 across the three treatment groups (including placebo) using a repeated measures ANOVA model including a treatment effect and a random time effect and adjusting for possible effects of investigational site and insulin use. Due to the size of this study relative to the number of factors under consideration, F-tests for main effects (treatment, insulin use, center, time) will be constructed ignoring possible interactions. Comparisons of active treatment group relative to placebo may be performed using a two-sided alternative.

In a secondary analysis, paired t-tests will be used to compare the baseline (Week 0) and Week 12 values of HbA_{1c} for each treatment group. Additionally, a repeated measure analysis, including terms for the fixed effects, treatment, insulin use, and center, and the random effect:time, may be implemented to assess change from baseline in HbA_{1c} over the 12-week treatment period.

4.1.5 Sample Size Estimation

Available trial data from each subject who received at least one dose of study medication will be used in the primary safety analysis. Using PASS2000 (NCSS Software), a total of 45 subjects (15 per group) will be enrolled into the study to ensure that approximately 39 subjects (13 per group) complete the study under the following sample size assumptions:

- A parallel group design is employed, with equal randomization between the groups.
- The primary endpoint is relative insulin response over time.
- The comparison is made using a Type I error rate of 0.05.
- The comparison is assessed using a Type II error rate of, at most, 0.2 which provides a minimum power to detect the specified difference 80%.
- An F-test from a repeated measures ANOVA model including a fixed treatment effect and a random effect for time is used to test the null hypothesis of no treatment difference.
- The baseline value for the placebo group is 0.23. A 15% difference is expected in each of the treatment groups. A constant standard deviation of 0.20 is expected[21].
- The relative insulin response is calculated at baseline, week 1, week 4, week 8, and week 12.

Similar calculations suggest that a sample size of 13 subjects per group will be sufficient to detect a 5% change from baseline in HbA_{1c} for each treatment group (including placebo) at week 12 under the following sample size assumptions:

- Each comparison is made using a Type I error rate of 0.05.

- Each comparison is assessed using a Type II error rate of, at most, 0.2 which provides a minimum power to detect the specified difference 80%.
- An effect size of 1 is expected.
- A paired t-test is used to test the null hypothesis of no treatment difference.

4.1.6 Randomization

Within each center, the investigator or his/her designate will determine whether the patient has met all inclusion/exclusion criteria and is eligible for entry into the study. Patients meeting all inclusion/exclusion criteria and providing written informed consent will be randomized at Visit 3, after completion of the lead-in period, which begins at Visit 2.

To maintain balance of treatment assignments among insulin use groups (insulin use, no insulin use), eligible patients will be randomly allocated in balanced blocks to either the TH9507 1 mg, TH9507 2 mg, or placebo treatment groups according to computer generated randomization codes for each insulin use group maintained by an external randomization center. To ensure that at least 3 subjects either in the insulin use or non-insulin use group complete the study for each treatment arm, enrollment for the larger group may be halted after 33 patients have been randomized for that group. Enrollment would continue for the smaller group until approximately a total of 12 subjects have been randomized for that group.

Study drug supplies will be assigned sequentially by the investigator, or his/her designate, within each center, using the subject randomization number assigned by the randomization center. Subjects may not be randomized into the study more than once, and once a subject randomization number has been assigned, it may not be reused.

Assignment of the randomization number to a subject defines enrollment into the study.

4.1.7 Blinding

The study is designed to be double-blind. Active drug and placebo will be provided as lyophilized powder in vials of identical appearance and supplied in blister cards covering a one month period at each monthly visit. Each dosage regimen is supplied as two vials according to the following ‘double-dummy’ specification:

- 1 mg TH9507 as one vial of 1.1 mg active drug and one vial of placebo
- 2 mg TH9507 as two vials of 1.1 mg of active drug
- placebo as two vials of placebo

The randomization code for each subject is supplied to the participating center in sealed envelopes that will only be opened in case of emergency. The study blind will be broken and treatment assignment will be made available for data analysis after the database has been locked and protocol violations have been determined.

4.1.8 Data Transformations

It is intended that the analysis of the primary endpoint will be based on relative insulin response. However, appropriate transformations will be performed as required to satisfy assumptions required to perform statistical analyses. Descriptive statistics will be provided for these transformed variables.

4.1.9 Strategy for Statistical Analysis

The safety population is defined as all subjects who were randomized and received at least one dose of study medication (1 mg TH9507, 2 mg TH9507, or placebo) during the course of the trial. This is the primary population for analysis.

The change from baseline values for biochemical laboratory parameters (secondary endpoints) will be analyzed using analysis of variance techniques (ANOVA) which focus on the between (different regimens) and within (same regimen) comparisons of these measurements over time, with center and insulin use group included as fixed effects. Analyses may be conducted as repeated measures analyses or individual analyses of specific study visits.

Glucose values will be obtained at home four times per day using a standardized glucose meter. Subjects will record glucose values in their diary. Glucose values, downloaded electronically from the glucose meters, will be used for statistical analysis.

The assessment of safety will be based mainly on the frequency of adverse events and on the number of laboratory values that fall outside of predetermined ranges. Other safety data (e.g. vital signs, special tests) will be considered as appropriate.

Adverse events will be summarized by presenting, for each treatment group, the number and percentage of subjects having any adverse event, having an adverse event in each body system and having each individual adverse event. Any other information collected (e.g. severity or relatedness to study medication) will be used to categorize the adverse event data as appropriate.

Laboratory data will be summarized by presenting shift tables using extended normal ranges (baseline to most extreme post-baseline value) by presenting summary statistics of raw data and change from baseline values (means, medians, standard deviations, ranges) and by flagging of notable values in data listings.

4.1.10 Interim Analyses

No formal interim efficacy analyses are planned.

4.1.11 Dropouts, Protocol Deviations and Exclusions

Subjects may end their participation in the study at any time for any reason(s). These subjects will be encouraged to provide a termination assessment at this time.

Patients screened for enrollment into the study but found to be ineligible will be excluded from entry. A log of exclusions, along with the reasons for exclusion, will be maintained. Ideally, this information will consist of the inclusion/exclusion case record form criteria for each excluded subject. The extent of the information collected will depend on the ethical guidelines within each center. This information can then be used to characterize the population being screened but excluded from the trial. Should enrollment problems be encountered during the trial, this information may indicate the primary reason(s).

All protocol deviations will be documented and categorized in the final clinical trial report according to standard classifications.

STANDARD PROTOCOL DEVIATION CATEGORIES

1. Entrance Criteria Not Met
2. Patient Received Incorrect Trial Medication
3. Concomitant Medication Violation
4 Missed Visit, Evaluation or Test
   - Missed Visit
   - Missed Evaluation
   - Missed Test
   - Missing Required Source Documentation
   - Missed Daily Record
   - Incomplete Daily Record
5. Incorrect Timing of Visit, Evaluation or Test
   - Incorrect Timing of Visit
   - Incorrect Timing of Evaluation
   - Incorrect Timing of Test
6. Informed Consent Signed After Entry of Subject into Trial
7. Trial Medication Non-Compliance
8. Incorrect Patient Number Assignment
   - Patient Number Assigned Out-Of-Order
9. Other

Examples of evaluations include physical examination and laboratory tests.

4.1.12 Handling of Missing Data

As the primary objective of this study is to assess the safety of the two doses of TH9507, no special handling of missing data are planned.

A secondary analysis for the primary endpoint will be assessed using the method of last-observation-carried-forward (LOCF) will be used to impute the data required for the assessment of the primary objective.

4.1.13 Multiple Significance Testing

Unless otherwise specified, all p-values will be reported to four decimal places using two-sided tests of significance with no correction for multiplicity (multiple outcomes or multiple comparisons) having been employed. Statistical significance will be assessed at the conventional level of 0.05 in all cases unless otherwise stated, and thus any significant findings for parameters other than the primary endpoint must be interpreted accordingly.

4.1.14 Multicenter Considerations

It is anticipated that each center will have the same capacity to enroll patients. As such, patients will be recruited concurrently in time and in equal numbers at each of the centers. Once the

number of centers is finalized, target enrollment figures for each center may be amended to this section of the protocol.

The primary endpoint will be based on central laboratory assessments of the blood samples collected from oral glucose tolerance testing. Statistical methods that can separate the investigator (center) effect from the treatment and insulin use group effects will be used. It is not anticipated that there will be sufficient sample size to estimate possible interactions.

4.1.15 Retrospective Power

No calculations of retrospective power are planned.

4.2 Data Management

4.2.1 Database Design and Creation

An appropriate database will be designed and created within a suitable database management system. This database will be designed to store the data as recorded on the case record forms (CRFs) and will ensure a one-to-one mapping between the CRFs and the electronic copy as stored in the system.

4.2.2 Data Coding

Adverse events will be coded using the MedDRA dictionary for adverse events while concomitant medications will be coded using the WHO medication dictionary. All other data will be coded according to usual numerical conventions in order to facilitate the data entry and verification process. Post hoc coding of all textual data will be undertaken wherever possible.

4.2.3 Data Entry and Verification

The data recorded on the CRFs will be entered via an appropriate database management system. The method of verification to be used is that of double data entry for all data. To facilitate the data entry and verification process, a data management manual will outline a set of guidelines for the review of the data. This document will:

- Identify specific data items to cross-reference and ensure consistency
- Establish data entry specifications
- Describe procedures for handling CRFs with missing or unclear information
- Establish acceptable windows for timing of events

4.2.4 Data Validation

After the data have been entered and verified, various edit checks will be performed for the purpose of ensuring accuracy, integrity and validation of the data base against the CRFs. These will include:

- Missing value checks
- Range checks
- Consistency checks
- Sequence checks

- Probabilistic checks
- Protocol adherence checks

Any inconsistencies that arise from these edit checks will be resolved with the site using Data Clarification Forms, which become part of the subject CRF.

4.2.5 Database Lock

On clinical completion of the study and after receipt of all outstanding CRFs (including Data Clarification Forms), the files will be locked after all data entry, verification and validation are complete and a database audit will be performed.

5. Reference List

1. Zbuzek V, Arnetz BB, Eneroth P, Zbuzek VK. Age-related differences in concomitant hormone release from the superfused rat adenopituitary. Gerontology 1991; 37:253-61.
2. Finkelstein JW, Roffwang P, Boyar RM, Kream J, Hellman L. Age-related change in the twenty-four-hour spontaneous secretion of growth hormone. J Clin Endocrinol Metab 1972; 35:665-70.
3. Ho KJ, Evans WS, Blizzard RM, et al. Effect of sex and age on the 24-hour profile of growth hormone secretion in man: importance of endogenous estradiol concentrations. J Clin Endocrinol Metab. 1987; 64:51-8
4. Shibasaki T, Shizume K, Nakahara M et al. Age-related changes in plasma growth hormone response to growth hormone-releasing factor in man. J Clin Endocrinol Metab 1984; 58:212-4.
5. Sonntag WE, Steger RW, Forman LJ, Meites J. Decreased pulsatile release of growth hormone in old male rats. Endocrinology 1980; 107: 1875-79.
6. Deslauriers N, Gaudreau P, Abribat T, Renier G, Petitclerc D, Brazeau P. Dynamics of growth hormone responsiveness to growth hormone-releasing factor in aging rats: peripheral and central influences. Neuroendocrinology 1991; 53:439-46.
7. Van cauter E, Leproult R, Plat L. Age-related changes in slow-wave sleep and REM sleep and relationship with growth hormone and cortisol levels in healthy men. JAMA 2000; 284:861-8.
8. Veldhuis JD, Iranmanesh A, Weltman A. Elements in the pathophysiology of diminished growth hormone in aging humans. Endocrine 1997; 7:41-8.
9. Abribat T, Deslauriers N, Reeves I, Garrel DR, Brazeau P. Alteration of growth hormone secretion in aging: peripheral effects. In: Growth Hormone II, Bercu BB and Walker RF Eds, Springer-Verlag Pub., 1994, pp155-66.
10. Hammerman MR. Insulin-like growth factors and aging. Endocrinol Metab Clin North Am 1987; 16:995-1011.
11. Florini JR, Prinz PN, Vitello MV, Hintz RL. Somatomedin C levels in healthy young and old men: relationship to peak and 24-hour integrated levels of growth hormone. J Gerontol 1985; 40:2-7.
12. Zachwieja JJ, Yarasheski KE. Does growth hormone therapy in conjunction with resistance exercise increase muscle force production and muscle mass in men and women aged 60 years or older? Physical Therapy 1999; 79:76-82.
13. Cummings DE, Merriam GR. Age-related changes in growth hormone secretion: should the somatopause be treated? Sem Reproductive Endo 1999; 17:311-26.
14. Cohn L, Feller AG, Rudman IW, Rudman D. Further observations on the effects of human growth hormone in elderly hyposomatomedinemic men. In: Growth Hormone II, Bercu BB and Walker RF Eds, Springer-Verlag Pub., 1994, pp133-49.
15. Cohn L, Feller AG, Rudman IW, Rudman D. Carpal tunnel syndrome and gynecomastia during growth hormone treatment of elderly men with low circulating IGF-1

concentrations. Clin Endocrinol 1993; 39:417-25.

16. Borst S, Lowenthal DT. Role of IGF-1 in muscular atrophy of aging. Endocrine 1997; 7:61-3.

17. Khorram O, Laughlin GA, Yen SC. Endocrine and metabolic effects of long-term administration of Nle27-growth hormone-releasing hormone (1-29)-NH2 in age-advanced men and women. J Clin Endocrinol Metab 1997; 82:1472-79.

18. Final Study Report, Protocol GRF-026C, Stability and metabolism of TH9507 in human plasma in vitro, September 1998, Theratechnologies Inc.

19. Final Study Report, Phase 1 crossover, randomized, and partially double-blind study to assess the pharmacodynamics and pharmacokinetics of a somatoliberine analogue (TH9507) and of hGRF (1-29)NH2 following single doses in healthy volunteers, 28 January 2000, Theratechnologies Inc.

20. American Diabetes Association: Clinical Practice Recommendations 2001. Report of the Expert Committee on the Diagnosis and Classification of Diabetes Mellitus. *Diabetes Care* 2001;24:S1. Available at http://journal.diabetes.org/Full Text/Supplements/Diabetes/Supplement101/S5.htm. Accessed March 12,2001.

21. Vaxilaire, M., Pueyo, M, Clement, K, Fiet, J, Timsit, J, Phillippe, J, et al. Insulin Secretion and Insulin sensitivity in diabetic and non-diabetic subjects with hepatic nuclear factor-1α (maturity-onset diabetes of the young-3) mutations. European Journal of Endocrinology 1999; 141: 609-18.
